# Supplementary material for: Photo-Crosslinked Coumarin-Containing Bis-Urea Amphiphile Hydrogels
Source: Gels. 2022 Sep 27;8(10):615. doi: 10.3390/gels8100615 (PMC9601853; doi:10.3390/gels8100615)
Supplement: Supplementary file 1 [file gels-08-00615-s001.zip › gels-1928011-supplementary.pdf]

gels

Supplementary materials

Photo-Crosslinked Coumarin-Containing Bis-Urea Amphiphile Hydrogels

Jie Liu [1], Xianwen Lou [1], Maaike J. G. Schotman [2], Patricia P. Marín San Román [1] and Rint P. Sijbesma [1,*]

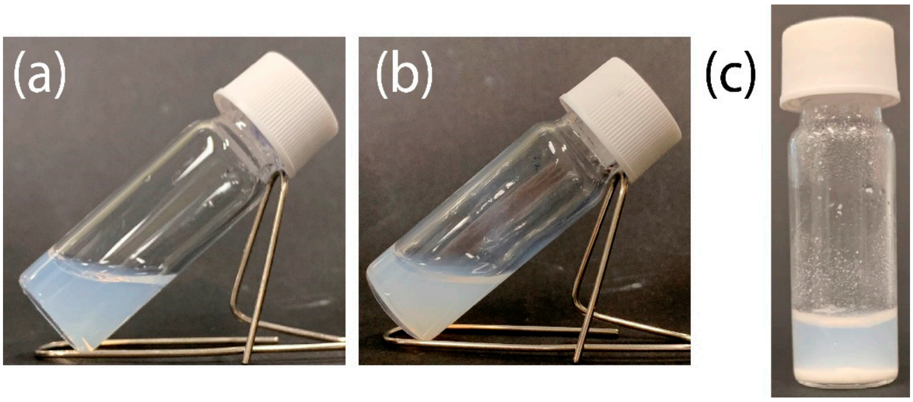

Figure S1. Photographs of 30 mg/mL OEG amphiphile (P8-10 OMe) solutions mixing with (**a**) 5%, (**b**) 10 % and (**c**) 20% coumarin based crosslinker, respectively.

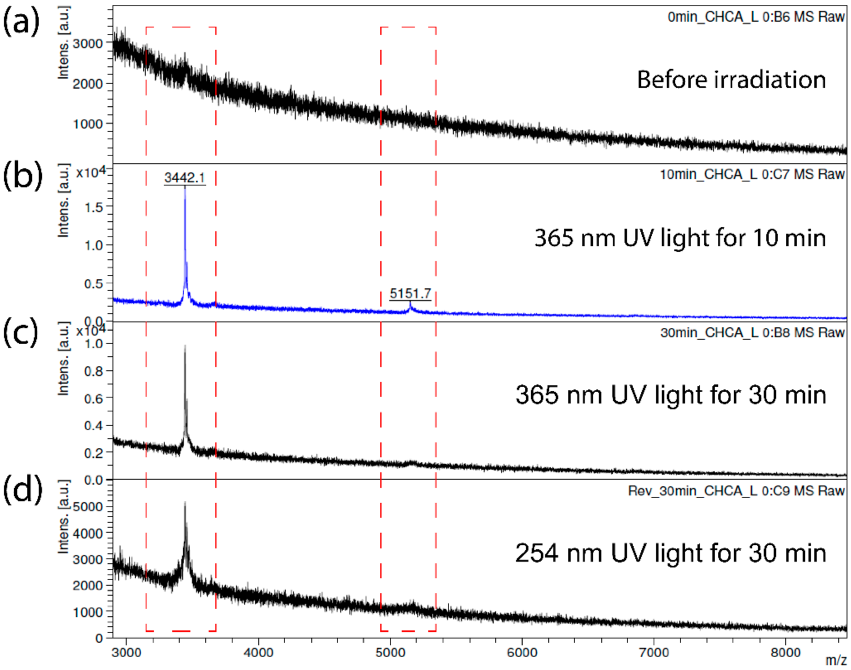

Figure S2. Zoom-in MALDI TOF MS results of OEG amphiphile solution containing 10% coumarin crosslinker before irradiation and after irradiation by UV light.
